# The Way to Decoding Pathogenesis and Conquering of National Afflictions, Viral Hepatitis and Liver Cancer

**DOI:** 10.31662/jmaj.2021-0099

**Published:** 2021-09-13

**Authors:** Kazuhiko Koike

**Affiliations:** 1 Department of Gastroenterology, Graduate School of Medicine, The University of Tokyo, Tokyo, Japan

**Keywords:** viral hepatitis, liver cancer, hepatitis B, hepatitis C, hepatocarcinogenesis, nonalcoholic steatohepatitis

## Abstract

Viral hepatitis and liver cancer are worldwide health problems, and they have been called national afflictions in Japan. We have been studying the mechanism of hepatocarcinogenesis, chiefly using experimental animal models, starting from clinical observations of patients. Observations of the early development of liver cancer in young patients with chronic hepatitis B with minimal hepatic inflammation and fibrosis, as well as the frequent development of hepatic steatosis in patients with chronic hepatitis C, prompted us to study direct roles of hepatitis viruses B and C in such liver pathogenesis. In addition, our establishment of a new paradigm, “hepatitis C as a metabolic disease,” further led us to elucidating the mechanism of nonviral, metabolism-associated liver cancer. Most hepatologists may appreciate a conquest in the war against viral hepatitis and associated liver cancer. However, even if we conquer the external enemies, hepatitis viruses B and C, an intrinsic enemy, metabolism-associated liver disease, would be waiting for us as an alternative cause of liver cancer. Confrontation of liver cancer has never ended.

## 1. Introduction

In Japan, approximately 30,000 people die of liver cancer annually. Because of the nationwide measures against viral hepatitis and liver cancer, which were conducted chiefly by hepatologists, death due to liver cancer peaked in 2005. The incidence of liver cancer declined after 2009. However, the 5-year survival rate of liver cancer (30%) is the third lowest, followed by pancreatic cancer and biliary tract cancer ^(1)^. Continuous measures are necessary to control hepatitis and liver cancer.

Causes of liver cancer include persistent infection with hepatitis B virus (HBV) and hepatitis C virus (HCV), alcoholic liver disease, and fatty liver disease due to overnutrition, along with some genetic disorders such as hemochromatosis. Among these, HBV and HCV infections are the major causes, and efforts have been concentrated on the elucidation of the pathogenesis of hepatitis and liver cancer and the development of therapies.

## 2. Epidemiology of Hepatitis B and C in Japan

The Japan Society of Hepatology (JSH), of which I had been Director General for 8 years, was established in 1965, the year of HBV discovery by Prof. Baruch S. Blumberg (Nobel laureate in 1976) ^(2)^. HCV was discovered in 1989, a quarter of the century after the birth of the JSH by Prof. Michael Houghton et al. (Nobel laureate in 2020) ^(3)^. Half a century after its discovery, HCV is now eradicated by the administration of oral tablets for as short as 8 weeks. HBV replication can be efficiently controlled with nucleot(s)ide analogs (NAs), although it has not yet been eradicated.

As of 2021, the rate of persistent infection (carrier rate) is approximately 1.0% for both HCV and HBV in Japan. The HBV carrier rate is 1.6% in Japanese people born in 1950, but it has decreased to 0.025% in those born in 1991, because of the arrangements of social environment, development of refined disposable medical equipment, and prevention of mother-to-child transmission through HB vaccination ^(4)^. Universal vaccination for HB was finally initiated in 2016. By preventing horizontal transmission, elimination of HBV from Japan is now coming into sight.

Most Japanese HCV persistent carriers contracted it in the turbulent period after the Pacific War II, far before the discovery of HCV. This explains the high carrier rate in the elderly and low carrier rate in the young in Japan ^(4)^. Unfortunately, the development of an effective HCV vaccine is not promising.

## 3. Exploring the Pathogenesis of Liver Cancer

**Figure 1.**
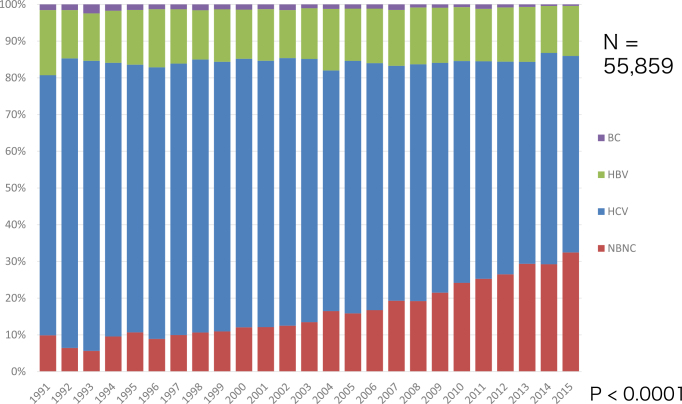
Changes in background liver diseases of liver cancer in Japan. Hepatitis C was the major cause, but it tended to decrease after 2004. Non-B, non-C liver cancer showed a rapid increase. HBV, hepatitis B virus; HCV, hepatitis C virus; NBNC, non-B, non-C.

In the past 30 years, HCV has been a major cause of liver cancer in Japan. HCV has received increasing attention because of its wide and deep penetration in the community, and it is associated with a very high incidence of liver cancer in persistent HCV infection. Once liver cirrhosis is established in hosts persistently infected with HCV, liver cancer develops at a yearly rate of approximately 7%-9%, resulting in liver cancer development in nearly 90% of HCV-associated cirrhotic patients in 15 years. By contrast, the rate of death due to HBV-associated liver cancer is constant at approximately 15% in the past 30 years, which is still a great threat to human health (Figure 1) ^(5)^. Globally, 400 million people are persistently infected with HBV, 170 million are infected with HCV, and viral hepatitis is the major cause of liver cancer. Recently, non-B, non-C (nBnC) liver cancer, which is defined as HBs antigen negative and HCV antibody negative, has been increasing to more than 30% of liver cancer cases ^(5)^. Alcoholic and nonalcoholic steatohepatitis (NASH) are considered to account for nBnC liver cancer, but the details are not yet clarified (Figure 1).

Several factors are thought to be associated with liver cancer development. Inflammation factors: Immune response to hepatitis virus causes death of hepatocytes, followed by their regeneration, leading to the accumulation of genetic aberrations including in oncogenes and tumor suppressor genes that confer a growth advantage to hepatocytes. Hepatitis viral factors: Viral proteins or genomes may exert direct oncogenic functions. For example, HBx of HBV may cause tumors in the liver, or the integration of the HBV genome may cause activation/inactivation of cellular genes. This factor is addressed in the following section. Genetic factors in the host: Single nucleotide polymorphisms of the HLA gene in HBV infection and MHC class I chain-related gene A in HCV/HBV infection may indicate susceptibility to liver cancer development. Environmental factors: Some toxins, such as aflatoxin B1, are responsible for liver cancer development. Metabolic factors: Obesity, insulin resistance (IR), diabetes, and fatty liver are known to be associated with liver cancer. Epigenetic factors: Epigenetic but not genetic alterations such as gene methylation or microRNA is known to be associated with liver cancer development.

Recently, next-generation genome sequencing studies on liver cancer revealed the existence of approximately 30 driver genes for liver cancer: Telomerase reverse transcriptase (TERT) overexpression, p53 mutations, and beta-catenin mutations are recurrent in human liver cancer associated with both hepatitis B and C ^(6)^. Although these are significant results, they also suggest that whole-genome/exome analyses do not reveal the whole design drawing of carcinogenesis in the case of liver cancer.

We demonstrated evidence of the direct involvement of HBV and HCV in distinctive ways in hepatocarcinogenesis. Our research plans started by targeting HBV, which was discovered ahead of HCV, inspired by the observation that liver cancer often develops in young patients with chronic hepatitis B who only had minimal liver inflammation and fibrosis.

## 4. Hepatocarcinogenesis in Hepatitis B: A Model of Viral Carcinogenesis

HBV is the first hepatitis virus belonging to the *Hepadnaviridae* family. The HBV genome consists of partly double-stranded circular DNA (3.2 kb) and replicates its genome through reverse transcription of mRNA using its own polymerase.

There is a strong association between the HBV carrier rate and incidence of liver cancer worldwide. A previous retrospective study in Taiwan indicated 223 times higher incidence of liver cancer in HBV carriers than in subjects without HBV infection ^(7)^. Moreover, a prospective study in Taiwan indicated that the viral loads of HBV at the time of study enrollment are a significant risk factor for future liver cancer development in HBV carriers ^(8)^.

### a) Oncogenic potential of the viral protein

**Figure 2.**
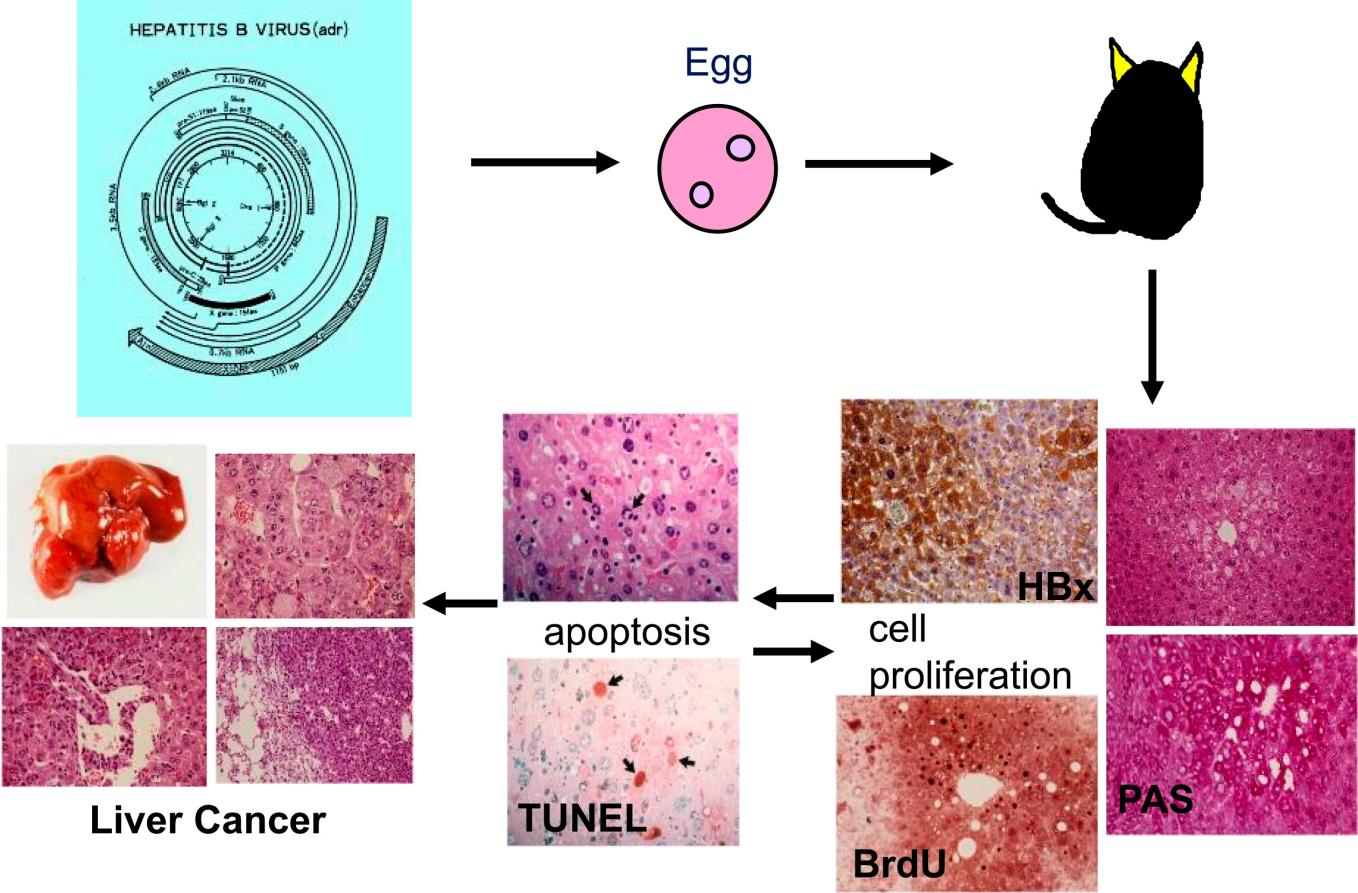
Estimated mechanism of hepatocarcinogenesis based on a hepatitis B mouse model. HBx functions as a transcription-type oncoprotein with both replicative and apoptotic functions. Additional secondary hits may accomplish *in vivo* carcinogenesis. PAS, periodic acid-Schiff staining; BrdU, bromodeoxyuridine uptake; TUNEL, TdT-mediated dUTP nick end labeling.

The X gene product (HBx) of HBV is suggested to be a viral factor involved in hepatocarcinogenesis. HBx is the transactivator of HBV and is a regulator of HBV replication. In the late 1980s, we hypothesized that a viral transactivator may act as an oncoprotein *in vivo* and examined whether HBx induces liver tumors by establishing transgenic mouse lines for the X gene. These transgenic mouse lines developed liver tumors, which were unusually malignant to metastasize to the mouse lung (Figure 2) ^(9), (10)^. HBx activates mitogen-activated protein kinase, leading to cell proliferation while also inducing apoptosis ^(11)^. In transgenic mice, it takes >12 months for the development of liver cancer, presumably after the acquisition of secondary hits (Figure 2). HBx is considered to exhibit carcinogenic action similar to transcription-factor-type oncogenes such as c-Myc or adenoviral E1A protein. By modulating cell proliferation/death of hepatocytes, HBx may be involved in hepatocarcinogenesis in hepatitis B.

### b) Integration of HBV genome into host genome

HBV has a remarkable characteristic that causes integration of its genome into the host when infecting the human liver. HBV integration is observed in 85% of liver cancer tissues from hepatitis B patients. Recent whole-genome studies have found HBV genome integrants near driver genes such as TERT, MLL4 and CCNE1 ^(6)^. It is of note that HBV integrants generally include the X gene, and the integrated X gene retains its transactivating function even with its 3’-end deleted. Thus, the integration of the HBV genome and HBx protein is the important viral factor in the development of liver cancer.

### c) Antiviral therapy and hepatocarcinogenesis

Currently, NAs and interferon (IFN)-alpha are used as antiviral therapy for HBV infection. Although HBV cannot be eradicated by either of two agents, clinical data indicate a reduction in the incidence of liver cancer development, particularly by the use of NAs, thus endorsing the importance of viral factors in the development of liver cancer.

## 5. Hepatocarcinogenesis in Hepatitis C: Distinct from Hepatitis B

HCV belongs to the *Flaviviridae* family, *Hepacivirus* genus, with a plus strand RNA genome of approximately 9,600 nucleotides. Once persistent HCV carriers enter the active hepatitis phase from the healthy carrier state, they rarely resolve spontaneously, with a high risk of progression to cirrhosis. It should be noted that in cirrhosis, liver cancer develops at a yearly rate of 7%-9% in hepatitis C.

Inflammation in the liver undoubtedly plays a pivotal role in the risk of liver cancer in patients with chronic hepatitis C. Patients with persistently higher serum ALT levels tend to develop liver cancer earlier than those with low serum ALT levels. However, it is notable that liver cancer is rare in autoimmune hepatitis, which is characterized by high serum ALT levels. The simple words “inflammation in the liver” do not rationalize the very frequent, multicentric hepatocarcinogenesis in chronic hepatitis C. Then, what additional mechanisms would allow such extraordinary mode of liver cancer development in hepatitis C?.

To answer this question, we established several mouse lines that are transgenic for some parts of the HCV genome cDNA, along with *in vitro* and culture cell experiments. Among the established mouse lines, the mouse lines transgenic the HCV core gene exhibited hepatic steatosis (fatty change) at a young age and finally developed liver cancer. In addition to elucidating the oncogenicity of HCV, we obtained lines of evidence that support a new paradigm, “hepatitis C as a metabolic disease” ^(12)^.

### a) Oxidative stress induction by HCV core protein

**Figure 3.**
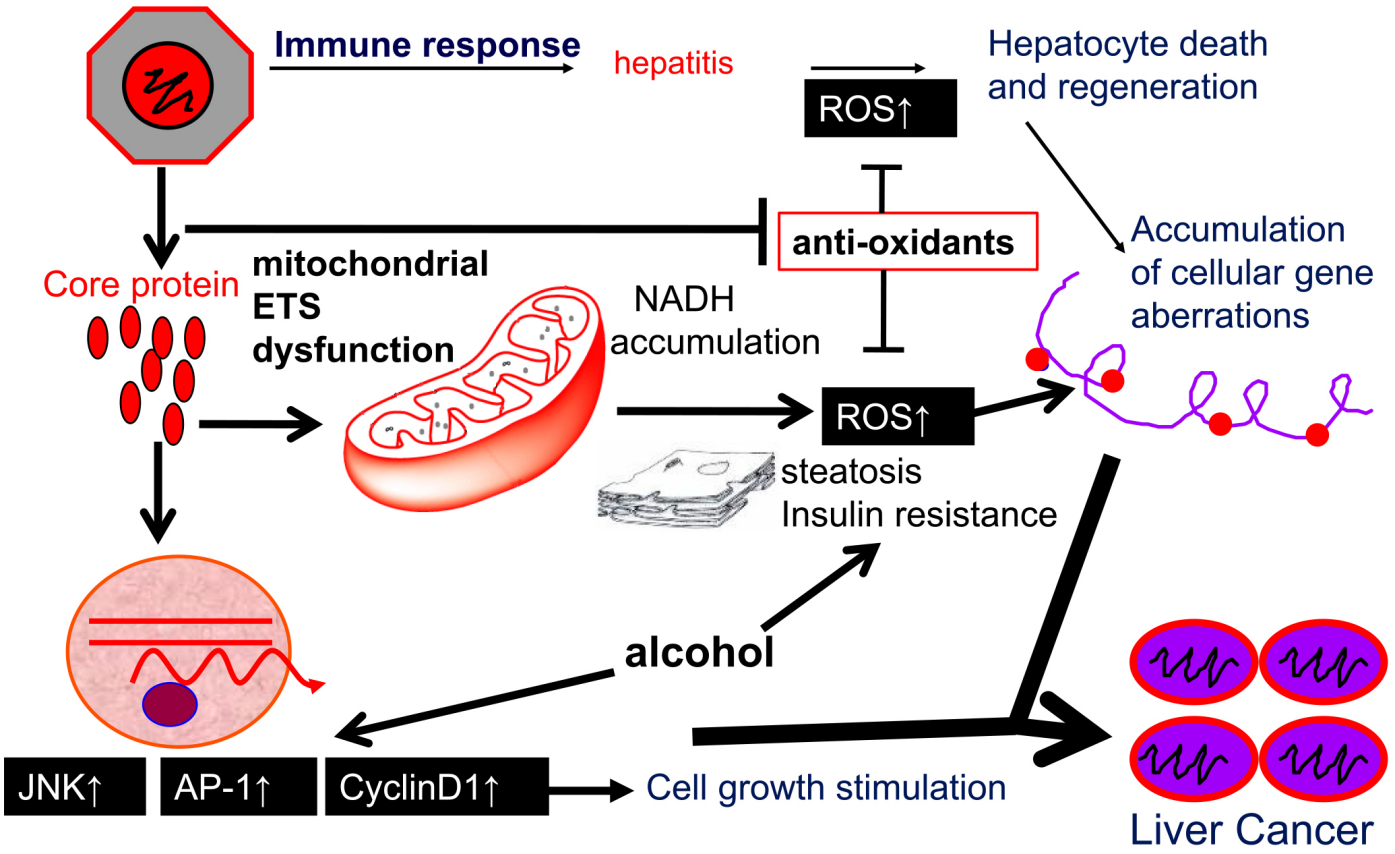
Molecular mechanism of liver carcinogenesis based on a hepatitis C mouse model. Induction of oxidative stress together with hepatic steatosis by hepatitis C virus core protein plays a pivotal role in the development of liver cancer. Alterations in cellular gene expressions, such as TNF-alpha, and those in the intracellular signaling pathways including JNK are co-accelerators of hepatocarcinogenesis in HCV infection. ROS, reactive oxygen species; ETS, electron transfer system; TNF-alpha, tumor necrosis factor-alpha; JNK, c-Jun N-terminal kinase; AP-1, activator protein-1.

The core protein of HCV induces liver cancer in transgenic mice in the absence of apparent inflammation ^(12)^. Their analysis revealed strong induction of reactive oxygen species (ROS) in the liver of the core gene transgenic mice compared with that of control mice, resembling human patients with hepatitis C, who suffer increased lipid peroxide in the serum and oxidative DNA damage in the liver ^(13)^. Detailed analysis revealed that the ROS originated from the mitochondrial electron system, where the functions of complexes I and IV are disturbed through the inhibition of the mitochondrial chaperone protein, prohibitin-1, by binding to the core protein ^(14)^. In addition, ROS are also induced by endoplasmic reticulum stress. Such overproduction of oxidative stress induces DNA damage and disturbs the stability of chromosomes, leading to the development of the bases for carcinogenesis (Figure 3).

### b) Modulation of intracellular signaling by HCV

The core protein also modulates intracellular signaling in hepatocytes. Activation of c-Jun N-terminal kinase by the core protein leads to the activation of activator protein-1, thereby strengthening the expression of cyclin D1 and cyclin-dependent kinase4, resulting in cell proliferation ^(15)^.

### c) Hepatic steatosis and hepatitis C

Our clinical observations of patients with hepatitis C indicated that hepatic steatosis was recurrent in patients with early chronic hepatitis C. The observation of hepatic steatosis in young transgenic mice for the core gene implies a direct effect of HCV on the induction of fatty liver, warranting further analysis ^(16)^. Currently, we understand that the activation/suppression of the following three pathways is responsible for the development of steatosis in hepatitis C. HCV activates the expression of sterol regulatory element binding protein (SREBP)-1c, which results in the overproduction of triglycerides. IR induced by HCV (see next section) increases the release of fatty acids from the peripheral tissues and its uptake into the liver. Disturbance of the activity of microsomal triglyceride protein (MTP) by HCV results in the inhibition of the secretion of very-low-density lipoprotein (VLDL) from the liver. Combination effects on the modulation of these pathways lead to the recurrent occurrence of hepatic steatosis in hepatitis C. It is notable that hepatic steatosis is an independent accelerating factor for fibrosis and liver cancer development in hepatitis C.

### d) Insulin resistance, diabetes, and hepatitis C

Hepatitis C patients occasionally develop IR before the development of cirrhosis. IR is reproducible in HCV core gene transgenic mice, indicating that HCV, in the absence of liver fibrosis or obesity, is responsible for the development of IR ^(17)^. An increase in inflammatory cytokines such as tumor necrosis factor-alpha is suggested to be a mechanism underlying IR. IR has also been demonstrated to be a significant independent determinant of the risk of liver fibrosis and cancer in hepatitis C. Eradication of HCV by antiviral treatment results in amelioration of IR in chronic hepatitis C patients. In addition, diabetes significantly increases the risk of liver cancer in hepatitis C.

### e) Progress in anti-HCV treatment and control of liver cancer

Antiviral therapy for hepatitis C starts with an IFN-alone regimen and has now progressed to an IFN-free direct antiviral agent (DAA) regimen. The rate of viral eradication (sustained viral response [SVR]) reached 95%-98% with a treatment duration of 8 weeks. The achievement of SVR significantly reduced the development of liver cancer. However, it should be noted that SVR is a cure for HCV infection but not for chronic liver disease. The risk of liver cancer development does not become nil, particularly in elderly, male hepatitis C patients with advanced liver fibrosis. Therefore, periodical medical consultations including liver imaging are indispensable long after the achievement of SVR.

## 6. Unanticipated Increase of Non-B, Non-C Liver Cancer in Japan

**Figure 4.**
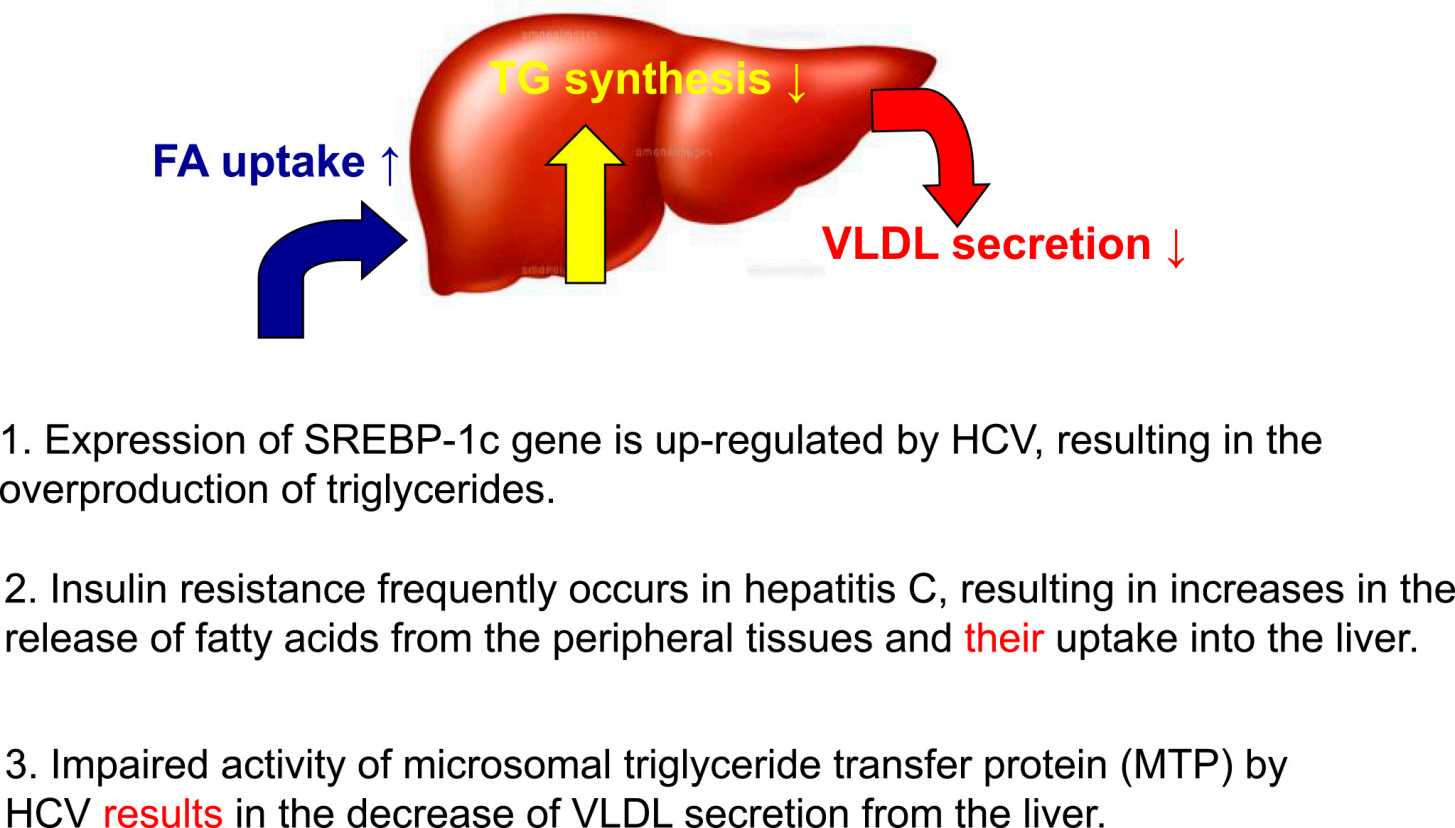
Mechanism of hepatic steatosis in hepatitis C. Hepatitis C virus (HCV) induces steatosis in the liver through the three pathways of lipid metabolism. First, a transcription factor, SREBP-1c, is upregulated by HCV core protein, resulting in an increased production of triglycerides. Second, HCV core protein induces insulin resistance, leading to an increase in the peripheral release and hepatic uptake of fatty acids. Lastly, the HCV core protein suppresses the activity of MTP, inhibiting the secretion of VLDL from the liver, yielding an increase of triglycerides in the liver. Thus, the involvement of three pathways easily leads to the development of hepatic steatosis in patients with hepatitis C. TG, triglyceride; FA, fatty acid; SREBP-1c, sterol regulatory element binding protein; MTP, microsomal triglyceride transfer protein; VLDL, very-low-density protein.

Although hepatitis B and C are still non-negligible causes of liver cancer, a significant increase in nBnC liver cancer, which has neither HBs antigen nor HCV antibody, is a recent big issue in Japan and worldwide. Our retrospective national survey data indicate that nBnC liver cancer accounts for more than 30% of all liver cancer cases (Figure 4) ^(5)^. What is nBnC liver cancer?

It is a heterogeneous disease group, mainly consisting of liver cancer associated with alcoholic and nonalcoholic fatty liver disease (NAFLD). In our detailed analysis of nBnC liver cancer ^(5)^, approximately 30% of nBnC liver cancers are associated with classical alcoholic cirrhosis. Another 30% of cases are associated with NAFLD or NASH. The remaining 40% of nBnC liver cancers are observed in obese/nonobese people who drink alcohol moderately (20< and <80 g/day), the details of which are currently under investigation. The comorbidity of diabetes is also a risk factor for nBnC liver cancer ^(18)^.

## 7. Conclusion

In this review, our data on the roles of HBV and HCV in the pathogenesis of chronic liver disease and liver cancer are described to conquer national afflictions, viral hepatitis, and liver cancer. The high rate of liver cancer development is caused by the combination of the hepatitis viruses themselves, inflammation mediated by immunity, metabolic factors, and environmental factors. High viral loads of HBV/HCV with continuous inflammation are significant risk factors for liver cancer. The conquest in the war against hepatitis and liver cancer may be closer because of the recent developments of DAAs for HCV and NAs and HB vaccination for HBV. However, an increase in nBnC liver cancer is prompt; hence, the development of measures against this is an urgent issue. Most hepatologists may praise the conquest in the war against viral hepatitis and associated liver cancer. However, even if we conquer the external enemies, HBV and HCV, an intrinsic enemy, metabolism-associated liver disease, would be waiting for us as a cause of liver cancer. Confrontation of liver cancer has never ended.

## Article Information

This article is based on the study, which received the Medical Award of The Japan Medical Association in 2020.

### Conflicts of Interest

None

### Sources of Funding

This work was supported by [AMED] grant numbers [JP20fk0210040] and [JP21fk0210090].
